# Expression of eosinophil β chain-signaling cytokines receptors, outer-membrane integrins, and type 2 inflammation biomarkers in severe non-allergic eosinophilic asthma

**DOI:** 10.1186/s12890-019-0904-9

**Published:** 2019-08-22

**Authors:** Virginija Kalinauskaite-Zukauske, Andrius Januskevicius, Ieva Janulaityte, Skaidrius Miliauskas, Kestutis Malakauskas

**Affiliations:** 1 Department of Pulmonology, Lithuanian University of Health Sciences, Kaunas, Lithuania; 2 Laboratory of Pulmonology, Department of Pulmonology, Lithuanian University of Health Sciences, Kaunas, Lithuania

**Keywords:** Severe non-allergic eosinophilic asthma, Eosinophil, IL-5, IL-3, GM-CSF, Integrins, Eotaxin, CCL17

## Abstract

**Background:**

Severe non-allergic eosinophilic asthma (SNEA) is a rare asthma phenotype associated with severe clinical course, frequent exacerbations, and resistance to therapy, including high steroid doses. The key feature is type 2 inflammation with predominant airway eosinophilia. Eosinophil maturation, activation, survivability, and recruitment are mainly induced by interleukin (IL)-3, IL-5 and granulocyte–macrophage colony-stimulating factor (GM-CSF) through their receptors on eosinophil surface and related with integrins activation states. The aim of the study was to estimate the expression of eosinophil β chain-signaling cytokines receptors, outer-membrane integrins, and serum-derived type 2 inflammation biomarkers in SNEA.

**Methods:**

We examined 8 stable SNEA patients with high inhaled steroid doses, 12 steroid-free patients with non-severe allergic asthma (AA), 12 healthy subjects (HS). Blood eosinophils were isolated using Ficol gradient centrifugation and magnetic separation. Eosinophils were lysed, and mRNA was isolated. Gene expressions of IL-5Rα, IL-3Rα, GM-CSFRα, and α4β1, αMβ2 integrins were analyzed using quantitative real-time reverse transcription polymerase chain reaction. Type 2 inflammation activity was evaluated measuring exhaled nitric oxide concentration (FeNO) collected with the electrochemical sensing device. Serum IL-5, IL-3, GM-CSF, periostin, chemokine ligand (CCL) 17 and eotaxin concentrations were assessed by enzyme-linked immunosorbent assay.

**Results:**

Eosinophils from SNEA patients demonstrated significantly increased gene expression of IL-3Rα, IL-5Rα and GM-CSFRα as well as α4, β1 and αM integrin subunits compared with the AA group. The highest IL-5 serum concentration was in the SNEA group; it significantly differed compared with AA and HS. GM-CSF serum levels were similar in the SNEA and AA groups and were significantly lower in the HS group. No differences in serum IL-3 concentration were found among all groups. Furthermore, serum levels of eotaxin, CCL17 and FeNO, but not periostin, differed in all groups, with the highest levels in SNEA patients.

**Conclusions:**

Eosinophil demonstrated higher expression of IL-3, IL-5, GM-CSF α-chain receptors and α4, β1, αM integrins subunits in SNEA compared with the AA group. Additionally, SNEA patients had increased serum levels of IL-5, eotaxin and CCL-17.

**Trial registration:**

ClinicalTrials.gov Identifier NCT03388359.

## Background

Asthma is a common lung disorder that usually lasts the sufferer’s lifetime. Despite the wide range of treatment options, in some cases, patients encounter a significant loss of quality of life, reduced work productivity, greater mortality risk, and higher health care resource use and costs [1, 2]. Severe asthma affects less than 10% of all asthma cases, but the treatment costs are high [3], disease severity is a major factor for clinical and economic burden [4]. This rare phenotype has sub-classifications driven by distinct pathophysiological processes. One of the severe asthma subtypes is a severe non-allergic eosinophilic asthma (SNEA), which is characterized by severe clinical course, frequent exacerbations of the disease, resistance to treatment including high doses of systemic glucocorticoids, and poorer clinical outcome [5, 6] as well as a good response to biologicals such as treatment with anti-IL-5 antibodies. The basis of this disease is eosinophilic airway inflammation, which plays a pivotal role in the pathogenesis of both allergic and non-allergic asthma, but the paths through which the genesis is initiated varies. In allergic asthma cases, Th2 cells mainly release prototype cytokines such as interleukin (IL) 4, IL-5 and IL-13, thereby stimulating type 2 immunity, which has high antibody titers and eosinophilia. Non-allergic asthmatics have increased numbers of type 2 innate lymphoid cells (ILC2s), that upon stimulation with the epithelial cytokines produce Th2-associated cytokines, including high amounts of IL-5, which is important for eosinophilic maturation and migration [7, 8]. This might explain severe eosinophilic inflammation in the absence of classical Th2-mediated allergy [9]. However, the eosinophilic properties themselves may be responsible for some eosinophilic inflammation features.

Eosinophils are circulating granulocytes that are produced via granulopoiesis in the bone marrow. IL-3, IL-5, and granulocyte-monocyte colony-stimulating factor (GM-CSF) are cytokines that are particularly important in regulating eosinophil development [10]. These three cytokines, also called eosinophilopoietins, are responsible for eosinophil maturation, activation, survivability, and apoptosis [10]. To recruit circulating eosinophils from the blood into the lungs, they need to be activated [11, 12]. For the most part, it depends on cytokines IL-3, IL-5, GM-CSF, as well as their receptors [11, 13]. The receptors for IL-3, IL-5 and GM-CSF each have unique α -chains but share a common β-chain. The β-chain is essential for signal transduction and explains the overlapping activities of these cytokines. The overexpression of these cytokines or their receptors can lead to excessive or aberrant initiation of signaling, which results in pathological conditions [14]. A marked increase in eosinophil count in the tissues and blood is observed in SNEA cases, but data are lacking on whether this is due to the quantitative values of these three major cytokines or the change in expression of the receptor on the eosinophil.

Another important part of the inflammatory process in the asthma pathogenesis is direct eosinophil adhesion to a target cell, which is relevant for eosinophil activation and functions*.* Integrins are cellular receptors that contain an α and a β subunits that regulate extravasation of eosinophils from the bronchial circulation to the airway wall and airspace. Such movement into the asthmatic lung depends on integrins on circulating eosinophils. Eosinophils tether in flow and roll on bronchial endothelial cells, integrins on rolling eosinophils become further activated because of exposure to cytokines, eosinophils arrest firmly to adhesive ligands on the activated endothelium, and eosinophils transmigrate to the airway in response to chemoattractants [15, 16]. α4β1 and αMβ2 are likely the two most important integrins that mediate eosinophil adhesion and movement [16]. There is evidence that a change in the integrin expression can attenuate eosinophil-induced airway smooth muscle remodeling in asthma [17].

Currently-used type 2 inflammation biomarkers are peripheral blood eosinophils, sputum eosinophil count, exhaled nitric oxide concentration (FeNO), immunoglobulin E (IgE), serum periostin concentration, but some of them have limitations [18]. Sputum eosinophil count is calculated only in large centres, tissue-specific, time-consuming test. FeNO is cheap, not specific, loses specificity in smokers. No clear association has been identified between IgE or allergy as a biomarker of treatment responses or clinical outcome. Periostin measurement is poor available, confounded by growth in childhood, pregnancy, and dental disease. There is an increasing need for useful type 2 inflammation biomarkers, controlled the recruitment of activated eosinophils from the bloodstream into tissues, like CCL11 (eotaxin), CCL17, with predictive and prognostic value for the progression of the disease in SNEA patients and their link with clinical treatments.

We hypothesized that differences exist in eosinophil biological properties during SNEA compared with non-severe AA. Therefore, it was important to investigate factors that reflect eosinophil activity like specific cytokines and their receptors as well as integrin expression. The purpose of our study was to evaluate the expression of the main eosinophilopoietins IL-3, IL-5 and GM-CSF receptors and integrin subunits α4, αM, β1, β2 at the surface of eosinophils. Therefore, we investigated serum levels of type 2 inflammation biomarkers as eotaxin, periostin, and CCL17 chemokines.

## Methods

### Study population

The research protocol was approved by the Regional Biomedical Research Ethics Committee of the Lithuanian University of Health Sciences (BE-2-13). The study was registered in the U.S. National Institutes of Health trial registry ClinicalTrials.gov with identifier NCT03388359.

The study included patients with SNEA, those who were free of steroid non-severe AA, and healthy subjects (HS), who comprised the control group. The participants were men and women between the ages of 18 and 50 years who signed written informed consent. The patients were recruited from the Department of Pulmonology, Hospital of the Lithuanian University of Health Sciences.

Inclusion criteria for the SNEA group were: asthma diagnosis for at least 12 months; non-allergic phenotype, approved clinically and with negative skin prick tests; peripheral eosinophil count ≥0.3 × 10^9^/l during the screening visit or ≥ 0.15 × 10^9^/l if with documented eosinophil count ≥0.3 × 10^9^/l in the 12-month period before the screening; no other reasons that could lead to poor control of asthma symptoms; documented at least 12-month treatment of high doses of inhaled corticosteroids combined with long-acting beta-agonist ± long-acting antimuscarinic agent ± episodic use of oral corticosteroids prior to inclusion in the study; in the 12 months before the screening visit ≥2 exacerbations of asthma that required treatment with systemic glucocorticoids.

The non-severe AA group comprised individuals with newly-established and untreated non-severe AA, approved with symptoms and medical history more than 1 year, positive skin prick test to clinically relevant allergen(s), and positive bronchial challenge with methacholine or bronchodilator reversibility test.

Healthy subjects should have been without allergic and other chronic respiratory diseases.

Exclusion criteria included asthma exacerbation ≤1 month prior to the study, clinically significant permanent allergy symptoms, active airway infection 1 month prior the study, use of oral steroids ≤1 month prior to study, and treatment with targeted (biological) therapy (e.g. omalizumab, mepolizumab, benralizumab).

All participants were neither current nor former smokers.

### Pulmonary function testing

The lung function was evaluated for all study subjects by measuring baseline forced expiratory volume in 1 s (FEV_1_), forced vital capacity (FVC), and FEV_1_/FVC ratio using a Ganshorn spirometer (Ganshorn Medizin Electronic, Germany) and compared with the standardized values according to investigated individual sex, age and height. Each of these values was measured three times and recorded only the highest of three reproducible measurements.

### Testing airway responsiveness

The airway responsiveness test was performed for AA patients and healthy individuals. Testing was performed using inhaled methacholine via a pressure dosimeter (ProvoX, Ganshorn Medizin Electronic, Germany). Aerolized methacholine was inhaled at 2-min intervals starting with a 10,1 μg methacholine dose and increasing it by steps until was achieved a 20% decrease in FEV_1_ from the baseline value either consumed the total cumulative methacholine dose of 1310 μg. Methacholine induced bronchoconstriction was expressed in percentage decrease of FEV_1_ from the baseline value. PD_20_ (methacholine dose causing a ≥ 20% fall in FEV_1_) was measured from the logarithmic dose-response curve by linear interpolation of two adjacent data points.

### Bronchial reversibility test

FEV_1_, FVC were registered before and 20 min after salbutamol administration (200 mcg by MDI) using a Ganshorn spirometer (Ganshorn Medizin Electronic, Germany). A positive response to a bronchodilator generally is defined as an increase of ≥12% and ≥ 200 ml as an absolute value compared with baseline in either FEV_1_ or FVC. A bronchial reversibility test was performed for one subject in the AA group because testing airway responsiveness was not possible due to observed bronchial obstruction at the screening visit.

### FeNO measurement

All study subjects underwent fractional exhaled nitric oxide (FeNO) analysis with an on-line method using a single breath exhalation and an electrochemical assay (NIOX VERO®, Circassia, UK), according to ATS-ERS guidelines. Patients inspired eNO-free air via a mouthpiece immediately followed by full exhalation at a constant rate (50 mL/s) for at least 10 s. The mean of three readings at the end of the expiration (plateau phase) was taken as the representative value for each measurement. Values of 25 ppb or higher were considered elevated according to ATS-ERS criteria.

### Skin prick test

Skin prick tests were used to measure study individuals sensitivity for suspected and/or the most common allergy-causing substances (*D. pteronyssinus*, *D. farinae*, cat and dog dander, five mixed grass pollen, birch pollen, and mugwort allergen) by using standardizes allergen extracts (Stallergenes S.A., France). Negative control was performed with diluent (saline) and positive control with histamine hydrochloride (10 mg/mL). Results of the test were evaluated 15 min after application. The skin prick test was considered appropriate when the mean wheal diameter was ≥3 mm.

### Eosinophils isolation

Sterile BD Vacutainer® tubes supplemented with EDTA was used for a collection of peripheral blood. Peripheral blood granulocytes isolated by high-density Ficoll (GE Healthcare, Finland) centrifugation and hypotonic lysis of erythrocytes. The whole blood was added on Ficoll layer and centrifuged at 600 g force for 30 min at room temperature. Lower layer collected and washed with sterile H_2_O and 2xPBS until no red erythrocytes colour were left.

Negative selection magnetic eosinophils isolation (Miltenyi Biotec, USA) were used to seperate eosinophils from granulocytes mixture according to the method shown previously [19]. Eosinophils were separated in 97% purity and at least 95% viability. Purity was confirmed with May–Grunwald–Giemsa staining and light microscopy; viability with automatic cell counter ADAM (Witec AG, Switzerland) based on propidium iodide staining.

### RNA isolation and real-time PCR

We isolated whole cells RNA by using the commercial miRNeasy mini kit (Qiagen, Valencia, CA) based on the manufacturer’s provided recommendations. RNA samples with high protein contamination (A260/A280 ratio below 1.8 or above 2.0) or with lower RNA concentration than 20 ul/ml were excluded from experiments. Real-time PCR was carried out by using a Power SYBR® Green RNA-to-C_T_™ 1-Step kit (Applied Biosystems, Foster City, CA) with Fast Real-Time PCR 7500 system in following conditions: reverse transcription – at 48 °C (30 min); activation of AmpliTaq Gold® DNA polymerase - at 95 °C (10 min); denaturation – at 95 °C (0.25 min); annealing - at 50–60 °C (0.5 min) depending by primers; extension - at 72 °C (0.5 min). Denaturation, annealing and extension were repeating for 40 cycles. Received qPCR data were analyzed by the comparative cycle threshold method. Data were normalized to 18S ribosomal RNA endogenous gene. Primers used to analyze gene expression are shown in Table 1.

**Table 1:** Primers of cytokine receptors and integrin subunits used in the qPCR analysis

Gene	Forward primer	Reverse primer
18S (reference)	5′-CGC CGC TAG AGG TGA AAT TC-3′	5′-TTG GCA AAT GCT TTC GCT C-3′
IL-5Rα	5′-AAT GAT CTT TTT CTA GGT AGA-3′	5′-CCT CTG GAG CTT GAG ATA-3′
IL-3Rα	5′-TTA AGC AGG CAC CTC TGT CC-3′	5′-CTG AGC CTT TGC TTT CAT CC-3′
GM-CSFRα	5′-GCA TTC CTC CTG ATC CCA GA-3′	5-CCT GGA GTC AAA CCT CAC ATT G-3′
αM	5′- CAG ACA GGA AGT AGC AGC TCC T-3’	5′- CTG GTC ATG TTG ATG AAG GTG CT-3’
β_1_	5′-GTG TGG CCC AAG ACA GTT CT-3’	5′-GGT TAC CCC ACC CTC TGA CT-3’
α_4_	5′- GCT TCT CAG ATC TGC TCG TG-3’	5′- GTC ACT TCC AAC GAG GTT TG-3’
β_2_	5′-AAC GTA TGC GAG TGC CAT TC-3’	5′-TTC ACG GGG TTG TTC GAC AG-3’

### Detection of protein level in investigating individuals blood serum samples

Protein (IL-5, IL-3, GM-CSF, periostin, chemokine ligand (CCL) 17 and eotaxin) levels in blood serum samples were measured by the enzyme–linked immunosorbent assay (ELISA) according to the instructions provided by manufacturers. ELISA kits used for experiments: IL-5 (Invitrogen, Carlsbad, California, US) LLD – 1.5 pg/ml; IL-3 (Invitrogen, Carlsbad, California, US) LLD- 1 pg/ml; Eotaxin (Invitrogen, Carlsbad, California, US) LLD-2.2 pg/ml; Periostin (Thermo Scientific, Waltham, Massachusetts, US) LLD – 80 pg/ml; CCL-17 (Thermo Scientific, Waltham, Massachusetts, US) LLD-5 pg/ml; GM-CSF (Affymetrix, Santa Clara, California, US) LLD-2.9 pg/ml. 100 ul of serum samples were used for experiments. Blood was collected into BD Vacutainer® Blood Collection Tubes and allowed to clot for 30 min. After that tubes were centrifuged at 1000×*g*, 10 min, 4 °C to remove the clot. The resulting supernatant is designated serum. Serum immediately collected and poured into 1 ml cryogenic tubes and frozen in − 80 °C for protein levels analysis. ELISA measurements were performed after the sufficient amount of samples was collected. The results were expressed as protein concentration per 1 ml of serum.

### Statistical analysis

Data statistics were performed by using the GraphPad Prism 6 (ver. 6.05, 2014; GraphPad Software Inc., San Diego, CA). Normally distributed gene expression data represented as the mean and standard error of the mean (SEM), protein concentration data as median [range]. Data statistics between independent groups were determined using the Mann–Whitney *U*-test, for dependent groups - the Wilcoxon matched-pairs signed-rank test. One sample t-test was used for statistical analysis with reference to theoretical mean. Results considered statistically significant when *p* < 0.05.

## Results

### Characteristics of the study population

We examined 32 nonsmoking adults (11 men and 21 women): 8 SNEA patients, 12 steroid-free non-severe AA patients, and 12 healthy controls. The SNEA group was distinguished not only by the increased blood eosinophil count, but also by age, lung function, and FeNO concentration (Table 2). The atopy component had only AA group patients. The IgE level was highest in the AA group and significantly differed from that in the SNEA group (*p* < 0.05).

**Table 2:** Demographic and clinical characteristics of the study population

	SNEA group patients	AA group patients	Healthy subjects
Number, n	8	12	12
Sex, M/F	1/7	7/5	3/9
Age, years	52.0^*#^ ± 4.2	26.0 ± 2.4	29.0 ± 2.2
BMI, kg/m^2^	25.5 ± 1.9	25.1 ± 1.8	22.5 ± 1.5
FEV_1_, l	1.5 ± 0.2^*#^	4.0 ± 0.2	3.9 ± 0.2
FEV_1_, % of predicted	56.6 ± 7.2^*#^	94.6 ± 3.8	103.8 ± 2.7
PD_20_, mean (range), mg (n) ^γ^	ND	0.11 ± 0.02	NR
Blood eosinophil count, × 10^9^/l	0.71 ± 0.17^*#^	0.33 ± 0.06^#^	0.20 ± 0.02
FeNO, ppb	59.3 ± 10.1^*#^	57.6 ± 5.5^#^	14.3 ± 1.4
IgE, IU/ml	128.3 ± 30.2^*#^	174.3 ± 26.5^#^	33.0 ± 7.1

*AA* Allergic asthma, *SNEA* Severe non-allergic eosinophilic asthma, *F* Female, *M* Male, *FEV*_*1*_ Forced expiratory volume in 1 s, *PD*_*20*_ The provocation dose of methacholine causing a 20% decrease in FEV_1_, *FeNO* Fractional exhaled nitric oxide, *IgE* Immunoglobulin E, *ND* Not done, *NR* Not responded

Data presented as the mean ± standard error of the mean

^γ^airway responsiveness was tested for 10 AA subjects; 1 subject had positive bronchial reversibility test

^*^p < 0.01 comparing with AA group

^#^p < 0.01 comparing with HS group

### Expression of β-chain cytokines receptors by eosinophils

**Fig. 1:**
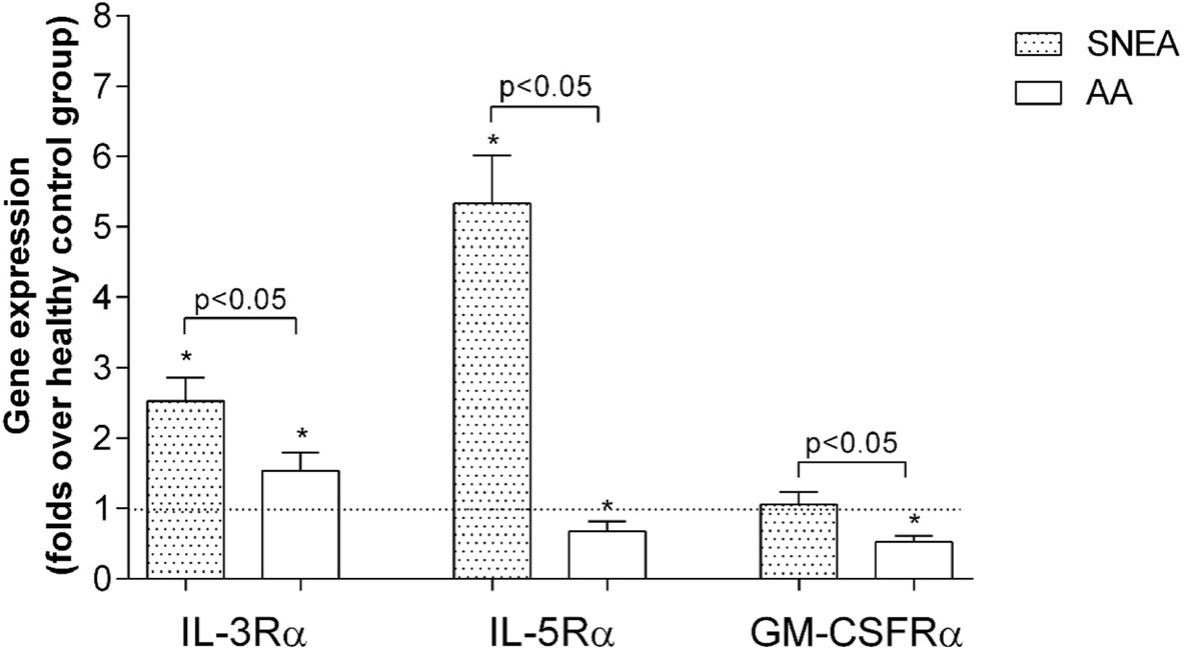
Genes expression of IL-3, IL-5 and GM-CSF receptors in eosinophils. Results presented as mean ± SEM. Severe non-allergic eosinophilic asthma (SNEA) group *n* = 8, non-severe allergic asthma (AA) group *n* = 12, healthy control group *n* = 12. *p < 0.05 comparing with healthy control group. Statistical analysis – Mann-Whitney *U*-test for analysis between SNEA and AA; One sample t-test for analysis against HS group

We investigated three main cytokines - IL-5, IL-3, and GM-CSF α-chain receptors gene expression, which are responsible for eosinophil activation, maturation, and survivability in eosinophils from SNEA, AA, and healthy individuals. We found that in SNEA group IL-3Rα and IL-5Rα gene expressions were significantly increased by 2.5 ± 0.3-fold and 5.3 ± 0.7-fold, respectively (*p* < 0.05), compared with healthy controls, whereas expression of GM-CSFRα did not differ (1.1 ± 0.2-fold, *p* = 0.56). Comparing the AA group with healthy control subjects, we estimated that IL-3Rα gene expression increased by 1.5 ± 0.2-fold (*p* < 0.05), but IL-5Rα and GM-CSFRα were significantly decreased by 3.1 ± 1.2-fold and 2.4 ± 0.4-fold, respectively (p < 0.05; Fig. 1). Furthermore, we found significant differences in gene expression between the SNEA and AA groups. Eosinophils in SNEA patients significantly increased the gene expressions of IL-3Rα, IL-5Rα and GM-CSFRα by 1.7 ± 0.2-fold; 8.0 ± 1.0-fold and 2.1 ± 0.3-fold respectively (p < 0.05).

### IL-3, IL-5, and GM-CSF serum levels

**Fig. 2:**
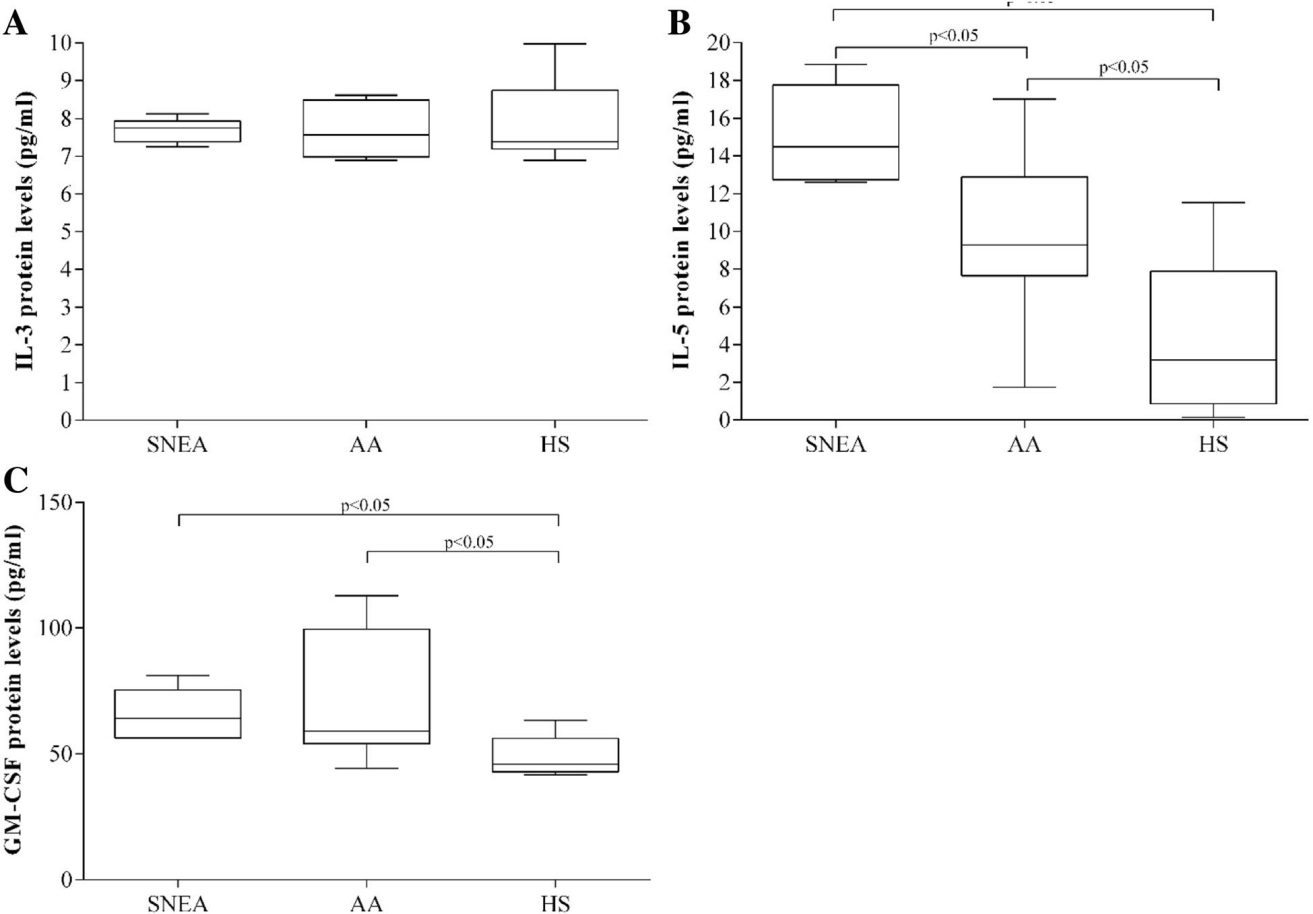
Serum levels of eosinophil maturation and activation cytokines. (**a**) IL-3 protein level. (**b**) IL-5 protein level. (**c**) GM-CSF protein level. Results presented as median (range). Severe non-allergic eosinophilic asthma (SNEA) group *n* = 5, non-severe allergic asthma (AA) group *n* = 11, healthy control group *n* = 10. Statistical analysis – Mann-Whitney *U*-test

IL-3 levels in serum did not differ among the SNEA, AA, and HS groups: it was 7.8 [7.3–8.1] pg/ml, 7.7 [6.9–8.6] pg/ml, and 7.9 [6.9–10.0] pg/ml, respectively (Fig. 2a). IL-5 levels were significantly higher in the SNEA group (14.5 [12.6–18.8] pg/ml) compared with the AA and HS groups (9.3 [1.7–17.03] pg/ml and 3.2 [0.2–11.5] pg/ml, respectively; Fig. 2b). No difference existed in GM-CSF levels in serum between the SNEA and AA groups (64.2 [56.1–81.2] pg/ml and 59.1 [44.2–112.8] pg/ml, respectively, *p* > 0.05); however, these levels were higher than in the HS group (45.91 [41.7–63.4] pg/ml; Fig. 2c).

### Eosinophil integrin expression

We investigated gene expression of two main outer-membrane integrins, α_4_β_1_ and αMβ_2_, by blood eosinophils, isolated from SNEA, AA, and healthy individuals. Both the SNEA and AA groups had significantly more mRNA of α4 (3.5 ± 0.7-fold and 1.8 ± 0.2-fold, *p* < 0.05), αM (10.2 ± 4.9-fold and 2.7 ± 1.3-fold, p < 0.05), β2 (2.5 ± 0.7-fold and 1.8 ± 0.3-fold, *p* < 0.05) and only SNEA group has increased β1 expression by 4.2 ± 2.0-fold compared with mRNA levels in healthy eosinophils (*p* < 0.05).

**Fig. 3:**
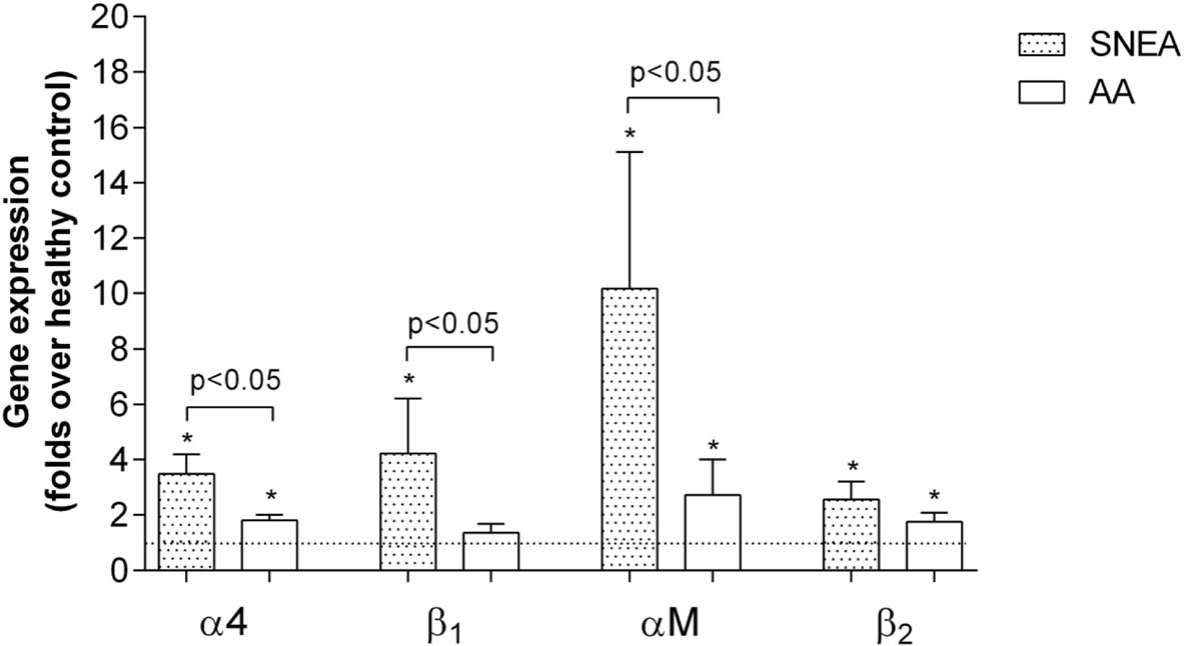
Gene expression of eosinophil integrins. Results presented as mean ± SEM. Severe non-allergic eosinophilic asthma (SNEA) group n = 8, non-severe allergic asthma (AA) group n = 12, healthy control group n = 12. *p < 0.05 comparing with healthy control group. Statistical analysis – Mann-Whitney *U*-test for analysis between SNEA and AA; One sample t-test for analysis against HS group

We revealed that eosinophils from the SNEA group had significantly more expressed α4, β1 and αM integrins subunits than those from the AA group (respectively by 2.1 ± 0.4-fold, 3.1 ± 1.5-fold, and 3.8 ± 1.8-fold, p < 0.05; Fig. 3).

### Type 2 inflammation biomarkers

**Fig. 4:**
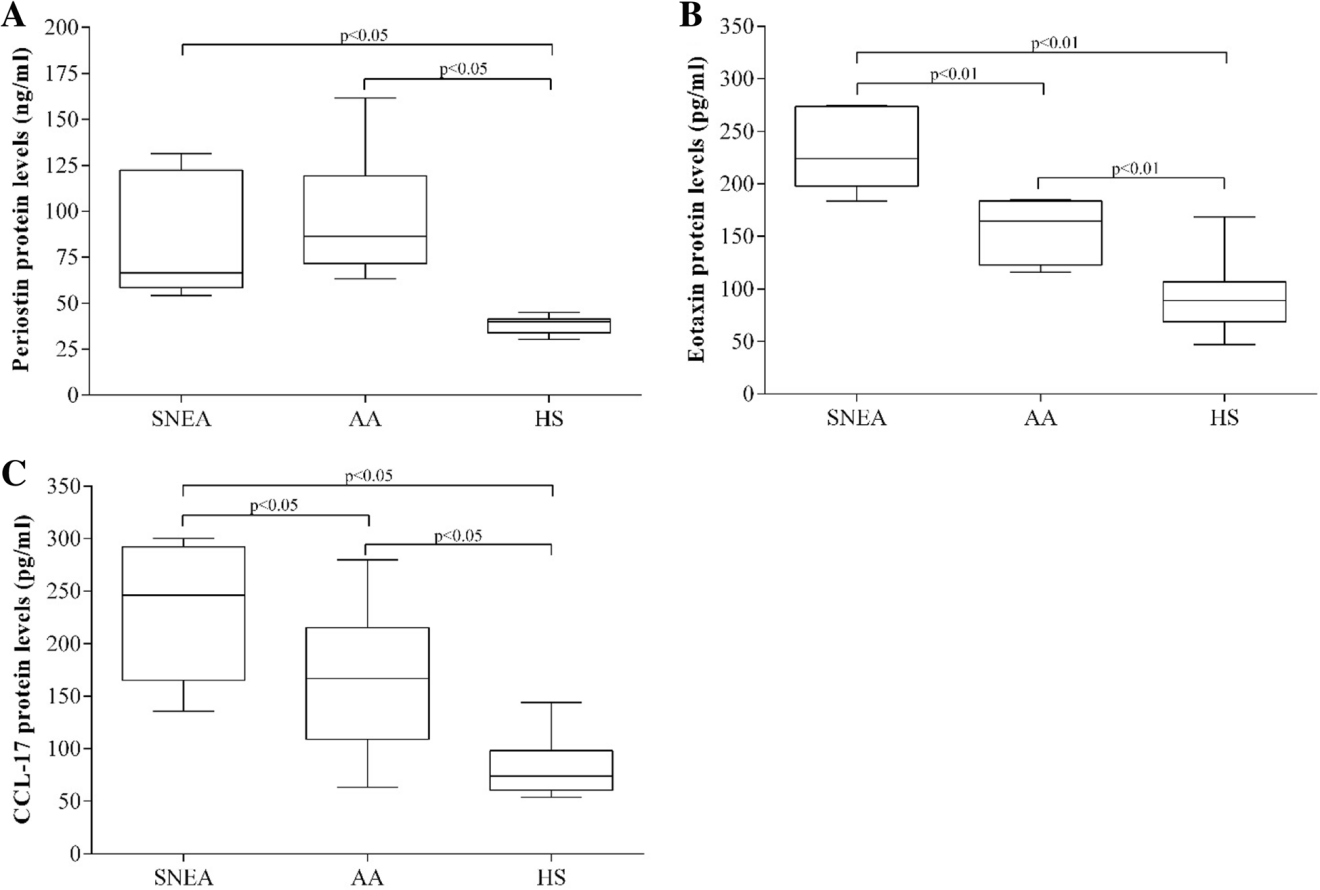
Serum levels of potential biomarkers for asthma severity. (**a**) Periostin protein level. (**b**) Eotaxin protein level. (**c**) CCL17 protein level. Results presented as median [range]. Severe non-allergic eosinophilic asthma (SNEA) n = 5, non-severe allergic asthma (AA) group n = 11, healthy control group n = 10. Statistical analysis – Mann-Whitney *U*-test

We investigated the levels of periostin, eotaxin and CCL17 serum levels in collected samples and observed the highest periostin levels in SNEA and AA groups, at 66.5 [54.2–131.5] ng/ml and 86.3 [63.3–161.7] ng/ml, respectively, without significant difference between these groups (*p* > 0.05). However, these levels were significantly higher than those in the HS group (40.0 [30.3–45.0] ng/ml, p < 0.05; Fig. 4a). The eotaxin level in the SNEA group was significantly higher than that in the AA and HS groups (224.2 [183.7–275.1] pg/ml, 164.7 [116.1–184.7] pg/ml, and 89.2 [47.1–168.5] pg/ml, respectively; Fig. 4b). Differences in CCL17 concentration in serum samples were similar to those in eotaxin levels - the highest CCL17 concentration was in the SNEA group (246.1 [135.6–300.4] pg/ml), medium in the AA group (167.0 [63.2–279.9] pg/ml) and lowest in the HS group (73.7 [53.7–144.2] pg/ml; Fig. 4c).

## Discussion

Several clinical parameters, like eosinophil count, decreased lung function, uncontrolled course and frequent disease exacerbations, and resistance to glucocorticoid treatment, help clinicians to differentiate severe and non-severe courses of asthma. However, in many cases, these clinical data could overlap or be insufficiently expressed, which limits clinicians’ ability to appoint appropriate medication. Therefore, new biological differences between asthma phenotypes should be revealed. In this study, we aimed to find which disease pathogenesis-associated biological aspects of eosinophils in SNEA patients could help to explain more severe disease course. We found that IL-3, IL-5, GM-CSF receptors, and α4, β1 and αM integrin subunit expression by eosinophils is increased in SNEA patients compared with non-severe AA patients. Moreover, IL-5Rα changes function from downregulation in AA patients to upregulation in SNEA patients. Furthermore, we identified higher eotaxin and CCL-17 serum levels, which in combination with validated eosinophilic inflammatory biomarkers could help to distinguish severe eosinophilic asthma, and predict the disease course, treatment effectiveness, and outcome.

Currently, the eosinophil count is the best-identified marker to determine eosinophilic asthma severity [20]. However, the number of eosinophils in patients with different types of asthma can overlap significantly and cause confusion among clinicians about appropriate treatment. Increased eosinophil count in peripheral blood is important, but not the only factor that contributes to disease pathogenesis because only pre-activated cells could significantly contribute to disease pathophysiological features. Eosinophil infiltration from blood to asthmatic airway depends on primed blood eosinophils, which leads to their arrest on activated endothelium, extravasation into the airway wall, and migration through airway tissues to the airway lumen. Eosinophil pre-activation is associated with maturation and activation mediators like β receptor-signaling cytokines IL-3, IL-5, and GM-CSF in the blood [21]. These eosinophilopoietins are responsible not only for eosinophil maturation in bone marrow but they also contribute to the regulation of eosinophils surface proteins, including integrins, intracellular adhesion molecule-1, cytokines receptors, L-selectin, CD-13 and others [22]. In our study, we measured mRNA levels of these cytokine α receptors in eosinophils in different investigating groups. The relationships between gene expression of these receptors and its ligands – IL-3, IL-5 and GM-CSF is not completely clear, confirming that signal transmission through receptor depends not only on ligand concentration but also on the number of receptors. IL-3 serum levels did not differ between investigating groups, but IL-3Rα gene expression in the SNEA group was significantly increased, which may show increased IL-3 signaling. GM-CSF serum level was similar in SNEA and AA patients but significantly increased compared with HS. However, the gene expression of GM-CSFRα in SNEA patients did not differ compared with healthy controls but significantly decreased in the AA group. This suggests that though GM-CSF serum levels were similar in SNEA and AA patients, its altered receptor expression shows increased GM-CSF signaling in SNEA patients. Intriguing results were observed regarding IL-5 concentration and its receptor expression. The highest serum concentration of IL-5 was in the SNEA group; this significantly differed from those in the AA and HS groups. IL-5Rα also increased dramatically in SNEA patients, while it significantly decreased in the AA group. This reveals a flip from downregulation to upregulation, which highlights the important role of IL-5 signaling in SNEA patients. However, recent therapeutic approaches targeting IL-5 alone have not completely ablated tissue accumulation of eosinophils and have had limited effects on disease progression, which suggests important roles for IL-3 and GM-CSF. β-chain cytokines change their roles at a different stage of disease development; moreover, expression of their receptors is regulated by different signaling pathways. Our result partly agrees with the works of Gregory et al., and Uchiyama et al., who investigated how IL-3, IL-5, and GM-CSF cross-regulate expression of these cytokine α receptors in healthy eosinophils [23, 24]. These teams revealed that IL-5Rα was down-regulated and IL-3Rα was upregulated by all β-chain cytokines, while GM-CSFRα was downregulated by GM-CSF, but was not affected by IL-3 or IL-5. However, the situation in asthma could be different due to complex pathogenesis interrupted by chronic inflammation conditions. Our results showed that GM-CSF could not downregulate its receptor expression in SNEA patients. More interestingly, according to Gregory et al., and Uchiyama et al., IL-5Rα expression in SNEA patients decreased dramatically. However, we found that IL-5 serum levels increased over 7-fold, but there was an important shift in IL-5Rα, with a visible significant increase in its mRNA levels by over 5-fold compared with healthy controls and 8-fold compared with AA. There is no clear explanation about these significant changes. As blood eosinophils count in SNEA patients is significantly increased compared with AA and HS, signaling through IL-5Rα might take an important part in enhanced eosinophilic inflammation and their survival. Increased mRNA levels of IL-5Rα could be associated with the soluble form of this receptor that can be generated by differential splicing of mRNA transcripts [25] or by cleavage of surface receptors [26]. Wilson et al. demonstrated that increased serum IL-5 levels and blood eosinophil count, as observed in our SNEA patients group, elevates soluble IL-5Rα [27]. Moreover, receptors expression is primarily controlled by specific transcription factors, which role in the regulation of eosinophils IL-5Rα transcription is almost unknown in asthma. Therefore, this altered process can lead to increased mRNA levels of IL-5Rα and different AA and SNEA pathogenesis. This may have implications with respect to the use of novel therapeutic agents targeting IL-5 and its receptor in patients with eosinophilia. However, IL-5 is recognized as an effective biomarker for other diseases in which eosinophilia is detected [28, 29]; therefore, its role as a biomarker for asthma severity is not yet defined.

Increased expression of eosinophil integrins is an important factor for more stable and enhanced adhesion mediating cell activation by interacting with counter-receptors on other cells or an extracellular matrix [15, 30]. Eosinophil expresses seven-transmembrane heterodimeric integrins, but α4β1 and αMβ2 receive the most important in asthma [15, 31]. Previously, enhanced expression of these integrins was identified in newly-diagnosed AA patients with the significant blocking effect of small tetrapeptide Arg-Gly-Asp-Ser (RGDS) [17]. Moreover, higher numbers of α4β1 and αMβ2 integrins in an activated state are related with better eosinophil arrest on endothelium cells that express elevated amount of vascular cell adhesion molecules-1 (VCAM-1) – the ligand for α4β1 [32] and intercellular adhesion molecule-1 (ICAM-1) for αMβ2 [33] thus contributing to increased eosinophilic airway inflammation. Although evidence exists about eosinophil integrin expression during AA, there are none about the expression of these integrins during SNEA. In our study, we found that expression of αM, α4, and β1 integrin subunits in eosinophils from SNEA patients significantly differ from AA patients. This suggests increased adhesive properties of these integrins required for enhanced infiltration into the lungs. Studies with integrin-deficient mice revealed that α4 and β2 integrins enhance eosinophil recruitment to the airways [34]. However, our study did not show significant β2 expression differences between SNEA and AA groups, which suggests that an amount of β2 subunit in eosinophils is sufficient and that only increased expression of the αM subunit is required for enhanced eosinophil activation through αMβ2 integrin, as was found in the SNEA group (Fig. 1). Additionally, Johansson et al. discovered that blood eosinophils stimulated with IL-5, IL-3, or GM-CSF specifically adhere and migrate to periostin via activated aMβ2 [35]. We confirmed that serum levels of periostin increased in SNEA and AA patients compared with healthy controls, as well as by IL-3, IL-5, and GM-CSF depending signaling in SNEA. However, because in SNEA only IL-5 levels and the expression of its receptors significantly increases, this may be the main trigger that activates aMβ2 integrins in eosinophils, leading to their enhanced recruitment into the lungs. Periostin, as a ligand for aMβ2, may act as an activator for eosinophils, but more important is their role modulating eosinophil transmigration, chemoattraction, and adhesion [36]. All these features could be activated in an autocrine manner because eosinophil is a predominant periostin producing cells in the blood [37].

Some overlap in asthma phenotypes can lead to an overlap in eligibility for the different biological therapies. Therefore, more specific biological markers should be identified which, together with well-known clinical properties, could help to separate SNEA in early-stage investigations. Blood eosinophil count is a good marker to identify eosinophilic asthma phenotype, together with sputum eosinophils count, which is the best-identified marker for airway eosinophilia. The number of eosinophils in sputum can be used as a marker of disease severity, with strong associations among airway eosinophilia, the severity of disease symptoms, and worsened lung function [38]. Unfortunately, sputum eosinophil count and their activity tend to be influenced by the current, mostly corticosteroid therapy and are not consistent, and airway eosinophilia could prevail in many asthma phenotypes. Asthma has a multicomponent pathology, and sputum eosinophil count alone does not disclose variability in asthmatics [39]. Currently, sputum eosinophil measurements are used only in places with limited clinical equipment because of its complexity and practical difficulties.

Many studies revealed the periostin as a significant marker to identify tissue eosinophilia, but our research data suggest that periostin alone is not suitable to identify asthma severity because of similar possible corticosteroid-affected periostin levels in SNEA and steroid-free AA patient groups*.* Moreover, periostin, as a marker, according to its expression mechanisms, is linked to anti-IL-13 [40] or anti-IgE treatment [40], but not with IL-5 associated biological therapies. FeNO is weakly affected by corticosteroid treatment and could be a more effective biomarker for asthma severity [41]. We tested our subjects and found significant differences in FeNO concentration among SNEA or AA and healthy control study groups. Increased FeNO level in the exhaled gas of asthma patients is linked to T-helper type 2 (Th2)-mediated inflammation [42]. FeNO strongly correlates with a sputum eosinophilia and lung functions and could help to identify eosinophil recruitment to asthmatic lungs. However, our data indicate that though differences in FeNO level exist between SNEA and AA groups, the disparity is insufficient to distinguish these two asthma phenotypes. This finding is in agreement with several reports that indicated the discordance between treatment response or adjustment and FeNO levels, especially after anti-IL-5 treatment [43–45].

In our study, eotaxin and CCL-17 protein act as the most selective markers because their levels in blood serum samples vary depending by asthma severity. One important study was made, indicating eotaxin as a potential diagnostic marker for asthma. Data analysis of thirty different studies revealed that eotaxin sputum level is a potential biomarker for asthma severity, but eotaxin serum level is also associated with lung function, sputum eosinophil count, eosinophil cationic proteins level, and FeNO [46]. Unfortunately, no anti-eotaxin approaches have been made so far. As Th2-mediated inflammation is associated with specific chemokines expression, like CCL-17 protein, by lung tissue cells for specific Th2 cell recruitment and activation, these chemokines in serum could act as specific biomarkers for disease severity. Significant correlations between CCL-17 and the severity of various diseases were published [47–49], highlighting its usage in allergic conditions, but no data exist on non-allergic asthma with predominant eosinophilia. We think that the release of eosinophilopoietins by CCL-17 activated Th2 cells leading to eosinophil maturation in SNEA patients reveals the role of CCL-17 as a potential biomarker. Recently was demonstrated that CCL17 and eotaxin are regulated by IL-4/IL-13 pathway and using anti–interleukin-4 receptor α monoclonal antibodies significantly reduce serum levels of eotaxin and CCL-17 [50]. Different concentrations of eotaxin and CCL-17 between SNEA and AA patients can be explained by Th2 cells, or ILC2 generated IL-4 and IL-13 cytokine levels, leading to altered activity of IL-4/IL-13 signaling pathway and its role in SNEA and AA pathogenesis.

There are some limitations to our study. First, a small number of patients with SNEA were investigated. However, this was sufficient to find significant differences between our study groups. Moreover, ICS treatment could also affect gene expression and protein levels, but to date, there is only evidence about the inhibitory effect of ICS that indicates that our received differences should be even higher. Also, almost all SNEA patients were older age women; consequently, it is necessary to assess the possible effects of ageing and sex on study objectives. To date, it has not been determined whether eosinophils differ by gender. Adhesion of eosinophils and chemotaxis upon eotaxin stimulation do not differ significantly between younger and older subjects [51]. However, no data show how eosinophil receptor expression changes with ageing. It has been noted that eosinophil degranulation in response to IL-5 stimulation is significantly decreased in older patients [51], but data were obtained not in a disease context. Furthermore, eosinophil receptor and integrin expression were possible to investigate only in mRNA levels that in rare cases, do not correlate with protein levels due to several parameters [52].

## Conclusion

We found that eosinophils in SNEA patients differ from those in AA because of increased gene expression of IL-3, IL-5, GM-CSFR receptors α-chains and α4, β1, αM integrins subunits, in conjunction with enhanced serum levels of IL-5. Moreover, increased serum levels of eotaxin and CCL-17 may become potential SNEA biomarkers.

## Data Availability

The datasets used and/or analysed during the current study are available from the corresponding author on reasonable request.

## Abbreviations

AA: Allergic asthma
ASM: Airway smooth muscle
CCL17: Chemokine ligand 17
DNA: Deoxyribonucleic acid
ELISA: Enzyme-linked immunosorbent assay
FeNO: Exhaled nitric oxide concentration
GM-CSF: Granulocyte-macrophage colony-stimulating factor
HS: Healthy subjects
IgE: Immunoglobulin E
IL-3: Interleukin 3
IL-4: interleukin 4
IL-5: Interleukin 5
IL-13: Interleukin 13
ILC2s: Type 2 innate lymphoid cells
PMN: Polymorphonuclear leukocytes
RNA: Ribonucleic acid
RT-qPCR: Real-time reverse transcription polymerase chain reaction
SEM: Standard error of the mean
SNEA: Severe non-allergic eosinophilic asthma
Th2: T helper 2 lymphocytes
